# Supplementary material for: Near-field imaging of surface-plasmon vortex-modes around a single elliptical nanohole in a gold film
Source: Sci Rep. 2019 Mar 29;9:5320. doi: 10.1038/s41598-019-41781-2 (PMC6441006; doi:10.1038/s41598-019-41781-2)
Supplement: Supplementary file 1 — Supplementary Info [file 41598_2019_41781_MOESM1_ESM.pdf]

Near-field imaging of surface-plasmon vortex-modes around a single elliptical nanohole in a gold film

Claudia Triolo[1*], Salvatore Savasta [1,2], Alessio Settineri[1], Sebastiano Trusso[3*], Rosalba Saija[1], Nisha Rani Agarwal[4], Salvatore Patanè[1]

[1] *Dipartimento di Scienze Matematiche e Informatiche, Scienze Fisiche e Scienze della Terra, University of Messina, Messina, Italy*
[2] *Theoretical Quantum Physics Laboratory, Cluster for Pioneering Research, RIKEN, Wako-Shi, Saitama, 351-0198, Japan*
[3] *CNR-IPCF, Istituto per i Processi Chimico-Fisici del CNR, Messina, Italy*
[4] *Faculty of Science, University of Ontario Institute of Technology, Oshawa, ON Canada*

*Corresponding authors: trusso@me.cnr.it, trioloc@unime.it

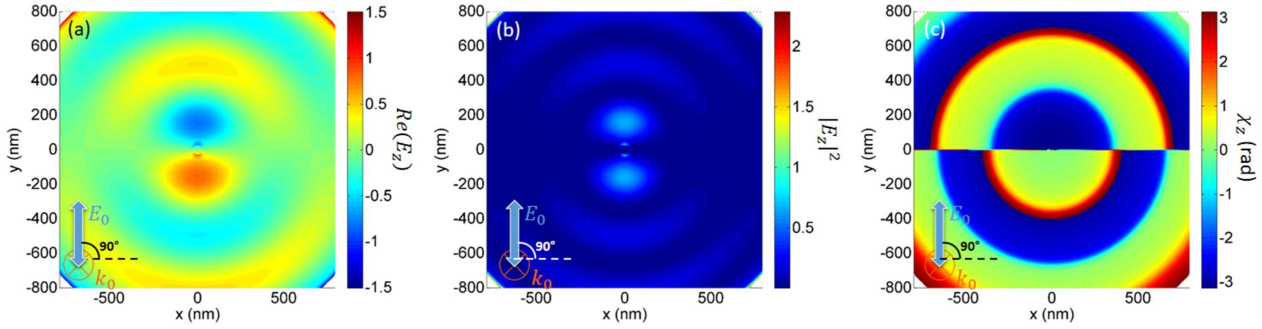

Figure S1. (a) Near-field intensity distribution of Re(E_z) around the circular nanohole, with a radius a=80 nm illuminated by a plane wave at λ_{exc}=632 nm, at 2 nm from the metal surface. Simulations are performed by finite element method (FEM) simulation. (c) Near-field intensity distribution of the *z*-component of the electric field $|E_z|^2$ around the circular nanohole illuminated by a plane wave at λ_{exc}=632 nm, at 2 nm from the metal surface. (d) Near-field intensity distribution of the total electric field $|E|^2$ around the circular nanohole illuminated by a plane wave at λ_{exc}=632 nm, at 2 nm from the metal surface.
